# Fanconi-like crosslink repair in yeast

**DOI:** 10.1186/2041-9414-3-7

**Published:** 2012-10-12

**Authors:** Danielle L Daee, Kyungjae Myung

**Affiliations:** 1 Genome Instability Section, Genetics and Molecular Biology Branch, National Human Genome Research Institute, National Institutes of Health, 49 Convent Drive, Bethesda, MD, 20892, USA

**Keywords:** Fanconi anemia, Interstrand crosslink repair, Mph1, Chl1, Slx4, Msh2, Msh6, Mhf1, Mhf2

## Abstract

Interstrand crosslinks covalently link complementary DNA strands, block replication and transcription, and can trigger cell death. In eukaryotic systems several pathways, including the Fanconi Anemia pathway, are involved in repairing interstrand crosslinks, but their precise mechanisms remain enigmatic. The lack of functional homologs in simpler model organisms has significantly hampered progress in this field. Two recent studies have finally identified a Fanconi-like interstrand crosslink repair pathway in yeast. Future studies in this simplistic model organism promise to greatly improve our basic understanding of complex interstrand crosslink repair pathways like the Fanconi pathway.

## Background

DNA damaging agents such as nitrogen mustard [1,2], formaldehyde [3], and cisplatin [4] generate many lesions that inhibit proper DNA replication and transcription. One such lesion, the interstrand crosslink (ICL), covalently links two complementary DNA strands and prevents their separation. Importantly, since both strands are damaged, an undamaged template strand is not available for repair. Due to these blocks and repair challenges, ICLs are considered one of the most toxic DNA lesions. It is estimated that the presence of just one unrepaired ICL is sufficient to kill yeast or bacteria [5] and approximately 40 unrepaired ICLs can kill mammalian cells [6]. As a result of this high cytotoxicity, crosslinking agents are common anticancer agents [7]. Outside of chemotherapies, ICLs can be induced by exposures in the environment [8] and byproducts of normal metabolic processes [9,10]. Thus, a clearer understanding of the mechanisms of ICL repair will inform our knowledge of both normal and cancer cells. This article and another recent review [11] describe novel findings in yeast that provide insight into the mechanisms of eukaryotic ICL repair.

### A yeast fanconi-like pathway emerges

Cells have the capacity to repair ICLs through highly complex DNA repair mechanisms. ICL repair in the prokaryotic system is relatively well defined. In *Escherichia coli*, nucleotide excision repair (NER) creates incisions on each side of the ICL. The resulting short oligonucleotide is attached through the ICL, but is displaced from the helix, revealing a gap. The gap is filled in by homologous recombination (HR) or translesion bypass synthesis (TLS), and the displaced oligonucleotide/ICL adduct is removed by NER [12].

In lower eukaryotes, defects in most known DNA repair pathways result in ICL sensitivity suggesting that eukaryotic mechanisms are much more complex, involve multiple repair pathways, and can occur in multiple phases of the cell cycle. Several recent reviews address this complexity in detail [13-23]. In the budding yeast *Saccharomyces cerevisiae*, a G1-specific repair pathway involves NER and TLS similar to the *E. coli* system [24]. Additionally, three independent epistasis groups (*PSO2*, *RAD52*, and *RAD18*) are implicated in ICL repair [25], but each pathway mechanism is not fully defined. Pso2 is an exonuclease that may be important for cleaving ICL repair intermediates [26-30]. HR proteins, including Rad52 and Rad51, likely fill in gaps post-incision and/or repair double strand breaks (DSBs) that arise during ICL repair. The post replication repair (PRR) pathway may help fill in the gaps after the incision and unhooking of ICLs.

In higher eukaryotes the Fanconi anemia (FA) DNA repair pathway has emerged as a master-regulator of downstream checkpoints and pathways of ICL repair [13]. This pathway was named for patients with the heritable, recessive disorder caused by mutations in FA repair genes. These mutations confer developmental defects, cancer predisposition, and marked sensitivity to ICL-forming agents [31]. In the FA repair pathway, FANCM and FAAP24 are thought to recognize blocked forks, activate checkpoint responses, and recruit the FA core complex (FANC A, B, C, E, F, G, L, FAAP100) [32-34]. FANCM is additionally stabilized by interactions with the MHF1/MHF2 complex [35,36]. After recruitment, the FA core complex ubiquitinates FANCD2 and FANCI [32,37]. These ubiquitinated proteins likely promote HR repair and other poorly understood downstream repair events mediated by FANCD1, FANCN, FANCP/SLX4, FANCO/RAD51C and/or FANCJ [13].

**Figure 1:**
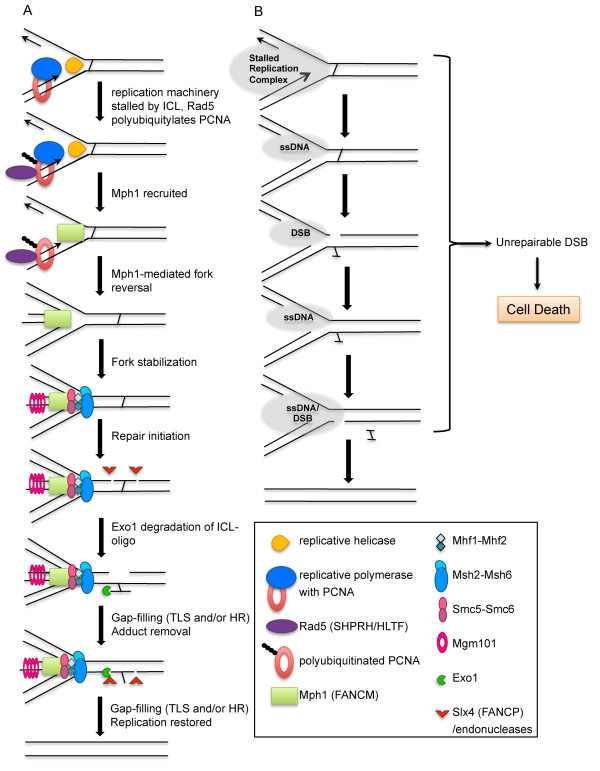
**Model for replication-associated interstrand crosslink repair in yeast.** (**A**) Replication is stalled by an ICL, Rad5 polyubiquitylates PCNA, and Mph1-mediated fork-reversal stabilizes the fork for repair (with Smc5/6 and Mhf1/2) and protects the repair intermediates from collapsing into double strand breaks (DSBs). Downstream events of repair are mediated by Slx4 and Exo1. HR and TLS are important for gap-filling steps. The figure key shows the putative human homologs in brackets. (**B**) The basic steps of ICL repair lead to various fragile intermediates (ssDNA, single strand DNA) that can collapse into DSBs. Cell death is triggered if the DSB cannot be repaired.

Studies in lower eukaryotic model organisms, like yeasts, have greatly improved our understanding of most DNA repair pathways. The single-celled yeast model is genetically tractable and provides a simplistic system for the study of complex DNA repair problems. Until recently, a yeast FA-like ICL repair pathway had not been functionally validated. Mph1, Mhf1/Mhf2, Chl1, and Slx4 are putative homologs to FANCM, MHF1/MHF2, FANCJ, and FANCP, respectively [34-36,38-41]. Although previous work established that the yeast proteins Mph1 [42-45], Mhf1/Mhf2 [35,36], Chl1 [46-48], and Slx4 [49] all play an important role in maintaining genomic integrity, a role in ICL repair (as indicated by mutant sensitivity to ICL agents) was not apparent. Recent publications from our group [50] and the McHugh group [51] have demonstrated that these proteins play a previously unappreciated role in ICL repair. Their function is important for ICL survival when either the Pso2 exonuclease or the PRR helicase Srs2 pathways are inactivated. These studies also revealed roles for additional proteins in the yeast FA-like pathway including Mgm101, MutSα (Msh2-Msh6), Exo1, proliferating cell nuclear antigen (Pol30/PCNA), Smc5/6 and Rad5. These studies provided key mechanistic insights that confirm, clarify, and bolster our knowledge of the FA pathway, allowing us to formulate the following model (Figure 1A):

ICL-induced replication stalling recruits or activates Rad5, which polyubiquitylates PCNA. The helicase Mph1 is recruited to reverse and stabilize the fork. Although their precise ICL-repair functions are unknown, Chl1, Mhf1/Mhf2, Smc5/6, and Mgm101 likely help stabilize Mph1 and/or the ICL repair intermediates. Slx4 may coordinate incisions surrounding the ICL with its associated endonucleases. Also in this pathway, the canonical mismatch repair complex Msh2-Msh6 (MutSα) potentially senses the aberrant DNA structure at the fork and/or recruits Exo1 to digest the tethered ICL-containing oligonucleotide. Oligonucleotide degradation produces a substrate for downstream processing events such as gap-filling by TLS polymerases or HR. Once the crosslinked adduct is removed, the DNA replication fork can be restored. Importantly, this reversed-fork pathway protects the fragile intermediates of repair (Figure 1B), which can collapse into double strand breaks and trigger cell death.

The foundational studies by our group and the McHugh group have validated the yeast FA-like pathway proteins [50,51]. Despite this, many questions remain about the precise functions of each protein, particularly Chl1, Smc5/6, and Mgm101. Chl1 and Smc5/6 have been implicated in sister chromatid interactions [52-54], so it is possible that these interactions create a stable intermediate for engagement by HR. Mgm101 has been implicated in mitochondrial recombination [55], so this role may extend to the nuclear compartment as well. Future genetic studies and the examination of ICL repair intermediates in different mutant backgrounds will hopefully shed light on these open questions.

In addition to the FA-like ICL repair pathway in yeast, Pso2 and Srs2 participate in ICL repair. The Pso2 nuclease functions after initial ICL recognition and incision, which is likely mediated by NER factors [56]. Srs2 is a helicase that directs the PRR pathway by preventing substrate engagement by recombination proteins [57,58]. Since PRR is a damage tolerance it is not clear how the ICL is excised through this pathway. It is entirely possible that, rather than forming independent pathways, the Pso2- and Srs2-mediated pathways represent the early (Pso2) and late (PRR) actions of a single pathway.

## Conclusion

Mechanistically, these studies confirm the existence of a yeast ICL repair pathway that is reminiscent of the mammalian FA pathway. Like the mammalian system [59], mismatch repair proteins contribute to the yeast FA-like pathway. These studies also clarify the controversial role of the PRR pathways [60-62] by demonstrating that, while the PRR proteins Srs2 and Rad18 are distinct from the FA pathway, Rad5 and PCNA are important mediators. Finally, in both yeast [50,51] and mammalian pathways [35,63], Mph1/FANCM-mediated fork regression or stabilization likely protects ICL repair intermediates from inappropriate processing or repair.

Despite the presence of a large core complex in the mammalian FA repair pathway, the yeast pathway appears to be substantially stripped down. It remains to be seen whether a core-like complex will be identified in yeast or whether evolutionary divergence was sparked by the need for the large complex in mammals. Furthermore, since the mammalian FA pathway appears to be a master regulator of repair it is surprising that the yeast pathway is secondary to Pso2- or Srs2-mediated events. Despite these differences, the simplified yeast model offers significant advantages for the FA repair field to address fundamental mechanistic questions in the future.

## Abbreviations

ICL: Interstrand crosslink; TLS: Translesion synthesis; NER: Nucleotide excision repair; HR: Homologous recombination; PRR: Postreplication repair; FA: Fanconi anemia; ssDNA: Single strand DNA; DSB: Double strand break.

## Competing interests

The authors declare no competing interests with the contents of this manuscript.

## Authors’ contributions

The manuscript was prepared by D.L.D with editorial and substantive advice from K.M. Both authors read and approved the final manuscript.
